# Innovative student placements at Northern Health

**DOI:** 10.1186/1757-1146-6-S1-P5

**Published:** 2013-05-31

**Authors:** Aspa Grollo, Anita Spring, Jana Gazarek, Ashley Morphet

**Affiliations:** 1 Northern Health, Melbourne, Victoria, 3076, Australia

## 

University course processes have recently changed with student enrolments increasing, ultimately placing new demands on student placements within the healthcare setting. In response to the changing university curriculum and in line with the Victorian Department of Health, Clinical Placement Networks and Health Workforce Australia, Northern Health (NH) Podiatry department identified a unique opportunity in provision of clinical placement education.

NH developed an innovative and exciting approach to the management and delivery of podiatry student clinical placements in order to increase capacity. Recruitment to a new Podiatry Clinical Educator (CE) position allowed implementation of strategies to significantly increase capacity, ensure provision of high quality, evidenced based and safe clinical education whilst supporting podiatrists and students.

To increase student capacity with limited resources, NH created a Podiatry CE position. This facilitated developments including; 2:1 Model of Supervision with peer learning and incorporating a focus on student feedback and reflection, integrating simulation into traditional clinical education, evidenced based tutorials and support to podiatrists and students.

Preliminary evaluation findings show significant increase in capacity with high student and podiatrist satisfaction. Results of student placement evaluations will be presented including perspectives on simulation, peer learning and specific podiatrist professional experiences.

With a Podiatry CE position and great team functioning; strategies implemented allow consistent provision of efficient, high quality, evidence based podiatry student clinical placements enabling increased capacity.

